# Crystal structure of the GDSL family esterase EstL5 in complex with PMSF reveals a branch channel of the active site pocket: Complex structure of GDSL esterase EstL5 with PMSF

**DOI:** 10.3724/abbs.2023108

**Published:** 2023-09-14

**Authors:** Runsha Chen, Xuechun Gao, Ting Nie, Jinhong Wu, Lin Wang, Ali Osman, Yan Feng, Xianghong Li, Yong Zhang

**Affiliations:** 1 School of Food and Bioengineering Changsha University of Science & Technology Changsha 410004 China; 2 State Key Laboratory of Microbial Metabolism Joint International Research Laboratory of Metabolic and Developmental Sciences School of Life Sciences and Biotechnology Shanghai Jiao Tong University Shanghai 200240 China; 3 Department of Food Science and Technology School of Agriculture and Biology Shanghai Jiao Tong University Shanghai 200024 China; 4 Gastro Endoscopy Center Shanghai Children’s Hospital School of Medicine Shanghai Jiao Tong University Shanghai 200062 China; 5 Biochemistry Department Faculty of Agriculture Zagazig University Zagazig Egypt

**Keywords:** GSDL esterase, PMSF, cavity, channel, crystal structure

## Abstract

Esterases/lipases from the GDSL family have potential applications in the hydrolysis and synthesis of important esters of pharmaceutical, food, and biotechnical interests. However, the structural and functional understanding of GDSL enzymes is still limited. Here, we report the crystal structure of the GDSL family esterase EstL5 complexed with PMSF at 2.34 Å resolution. Intriguingly, the PMSF binding site is not located at the active site pocket but is situated in a surface cavity. At the active site, we note that there is a trapped crystallization solvent 1,6-hexanediol, which mimics the bound ester chain, allowing for further definition of the active site pocket of EstL5. The most striking structural feature of EstL5 is the presence of a unique channel, which extends approximately 18.9 Å, with a bottleneck radius of 6.8 Å, connecting the active-site pocket and the surface cavity. Replacement of Ser205 with the bulk aromatic residue Trp or Phe could partially block the channel at one end and perturb its access. Reduced enzymatic activity is found in the *EstL5 S205W* and *EstL5 S205F* mutants, suggesting the functional relevance of the channel to enzyme catalysis. Our study provides valuable information regarding the properties of the GDSL-family enzymes for designing more efficient and robust biocatalysts.

## Introduction

Lipolytic enzymes are classified as serine hydrolases, including esterases, lipases and phospholipases, which are important biocatalysts because many of these enzymes not only catalyze specific hydrolysis in aqueous environment but are also able to conduct synthetic or transesterification reactions in organic solvents [1]. A number of lipolytic enzymes from various sources, with diversified enzymatic properties, have been isolated, characterized and widely used in chemical, pharmaceutical and food industries [ 1, 2] . In contrast to lipases which prefer water-insoluble triglycerides of long-chain fatty acids, esterases prefer short-chain ester substrates [3].

Many lipolytic enzymes hold the characteristic α/β-hydrolase fold in their tridimensional structure, mostly adopting the classical Ser-His-Asp catalytic triad and the oxyanion hole mechanism as well as holding a highly conserved G-X-S-X-G pentapeptide with Ser as the key catalytic residue [4]. However, GDSL family enzymes, as a subclass of lipolytic enzymes, share obviously distinct sequence motifs, having a conserved GDSL pattern close to the N-terminus of the protein [5]. Homologues of the GDSL family have been found from prokaryotes to eukaryotes and are involved in various biological processes, such as bacterial virulence, plant development and defense mechanisms [5]. Moreover, GDSL family enzymes are usually featured with four strictly conserved residues, Ser, Gly, Asn and His, in four conserved blocks, I, II, III and V, respectively; thus, this subclass of lipolytic enzymes is further classified into the SGNH hydrolase superfamily [6]. Structurally, the SGNH-superfamily members share a conserved three-layered α/β/α protein fold, which is different from the α/β-hydrolase fold. Moreover, some GDSL enzymes have been shown to have functional plasticity for catalyzing different chemical reactions. Indeed, GDSL enzymes usually have a structurally flexible active site that appears to change conformation in the presence and binding of different substrates, similar to the induced fit mechanism [6]; therefore, conformational dynamics might significantly affect the catalytic efficiency of GDSL enzymes.

We previously characterized a highly active GDSL family esterase, EstL5, from a thermophilic *G*. thermodenitrificans strain [7]. As a thermostable and organic solvent-tolerant esterase, EstL5 has potential industrial applications. Although the molecular mechanisms of GDSL enzymes have been reported [ 5, 6] , precise information on the catalytic features of GDSL esterases remains elusive. Because phenylmethanesulfonyl fluoride (PMSF) can covalently bind to and alkylate the hydroxyl of serine residues in the active site, it has been used as a potent inhibitor for serine hydrolases [8].

In the present study, we determined the structure of the EstL5-PMSF complex. Interestingly, the PMSF binding site is not located at the active site pocket but is situated in a surface cavity. Further structural analysis identified a long channel connecting the active site pocket and the surface cavity. Based on the structure guide, we tried to block the enzyme’s access channel with a bulk residue substituent and found that the resultant mutants, both *EstL5 S205F* and *EstL5 S205W* had reduced catalytic activities. Our study sheds new light on the catalytic properties of GDSL enzymes and offers scaffolds for the design of more efficient and robust biocatalysts.

## Materials and Methods

### Cloning, protein expression, and purification

*EstL5* without the deduced signal peptide sequence was cloned into the pET28b vector (Novagen, Gibbstown, USA), and the N-terminal His-tagged EstL5 recombinant proteins were expressed in *Escherichia coli* BL21 (DE3) cells and purified as described previously [7] The mutants were created by a modified QuickChange TM site-directed mutagenesis method with corresponding primers and a previously constructed pET-EstL5 plasmid as a template. All the mutants were verified by sequencing and then expressed and purified using similar procedure as for the wild-type (WT) EstL5 [7].

### Crystallization, X-ray data collection and structure determination

The EstL5-ligand complex crystals were prepared and optimized by the cocrystallization of PMSF (Thermo Fisher Scientific, Waltham, USA) and the purified EstL5 P184A/F185A protein (12 mg/mL) in 0.01 M CoCl _2_-6H _2_O, 0.1 M sodium acetate (pH 4.8) and 1.0 M 1,6-hexanediol. The molar ratio of protein to PMSF was 1:1.5. The single diamond-shaped crystal was directly frozen in liquid nitrogen after a brief transfer into a cryoprotection solution containing reservoir solution supplemented with 20% (v/v) glycerol and 2.0 mM PMSF. High-resolution diffraction data sets were collected at Shanghai Synchrotron Radiation Facility beamline BL18U with an ADSC Quantum 315r CCD areadetector (Shanghai, China). The collected data were processed and scaled with the XDS suite [9]. Phases were obtained by molecular replacement by using the CCP4 program phaser [10], with our previously determined EstL5 structure (PDB code: 7E16) as a template. The quality of the final structure was verified by the CCP4 program PROCHECK [11], which showed good stereochemistry according to the Ramachandran plot ( Table 1). The coordinates have been deposited in the Research Collaboration for Structural Bioinformatics Protein Data Bank with the accession code 8IK1 for EstL5-PMSF. All structural model figures were prepared by using PyMOL ( http://www.pymol.org).

**Table:** **Table 1** Data collection and refinement statistics for EstL5-PMSF cystal structure

Parameter	EstL5-PMSF
Data collection statistics	
Space group	*P*4 _3_2 _1_2
Unit cell parameters (Å)	105.153 105.153 128.001
Resolution range (Å)	2.47–2.35
Measured reflections	33256
Unique reflections	4241
Completeness	96.9
Redundancy	7.4
Average I/r(I)	2.05
Rmerge (%)	0.573
Refinement statistics	
Refinement resolution	29.51–2.35
Rwork (%)	0.2083
Rfree (%)	0.2438
r.m.s. deviation	
r.m.s.d. bond (Å)	0.007
r.m.s.d. angle (°)	0.907
Ramachandran plot (%)	
Most favored	96.25
Additionally allowed	3.75

### Mass spectrometric analysis

Protein crystals were picked out, washed and redissolved in 20 mM Tris-HCl buffer (pH 8.0). Protein samples were processed with 1 ng/μL trypsin solution (Promega, Madison, USA) overnight and loaded for LC separation on a nanoflow HPLC Easy-nLC 1200 system (Thermo Fisher Scientific). Digested peptides were analyzed on a Q Exactive plus mass spectrometer (Thermo Fisher Scientific). MS parameters were set as follows: ESI+ mode was used for full scan acquisition (m/z 350‒1800) in an orbital trap with a resolution of 70,000 (AGC 3e6) in data-dependent scan mode. The capillary temperature was 275°C, and the spray voltage was 2200 V. The daughter ions were measured on an orbit with a resolution of 17500 (AGC 1e5). The maximum fill times were set at 50 ms and 45 ms for the full scan and MS-MS scan, respectively.

### Access channel analysis

The access channels were analyzed by Caver Analyst 3.0 [12], with the following parameters: probe radius of 1.0 Å, shell depth of 4 Å, shell radius of 3 Å, and clustering threshold of 3.5. The simulations were conducted using the AMBER14 package with an ff12SB force field. The integration time step was set at 2 fs. The system was minimized with 500 steps of the steepest descent method and 3500 steps of the conjugate gradient algorithm and then heated to a finite temperature of 298 K in the NVT (constant particle number, volume and temperature) ensemble. After 10-ps simulations for equilibration, the simulations were processed in the NPT (constant particle number, pressure, and temperature) ensemble.

### Determination of biochemical and biophysical properties

The standard assay for esterase activity was performed at 30°C in 20 mM Tris-HCl buffer (pH 8.0) in the presence of 1.0 mM p-nitrophenol butyrate, and the reactions were initiated by adding 3 μL enzyme solution (0.1 mg/mL). After 5 min of incubation, the absorbance at 405 nm was measured. Enzyme kinetic parameters were calculated by measuring the enzyme activity at different concentrations (0.1 mM, 0.2 mM, 0.4 mM, 0.6 mM, 0.8 mM, 1.0 mM, 1.5 mM and 2.0 mM) of p-nitrophenol butyrate substrate and performing Michaelis-Menten equation analysis with GraphPad Prism 8.0.2 software. Differential fluorescence scanning (DFS) [13] was performed on a fluorescent quantitative PCR instrument (qTower Touch; AnalytikJena, Jena, Germany) at a heating rate of 1.0°C/min over a temperature range from 25°C to 90°C. The fluorescent dye 100× SYPRO Orange was mixed with 19 μL recombinant protein (0.3 mg/mL) to reach a final volume of 20 μL. The same amount of buffer was measured as a baseline, which was subtracted from each scan. The midpoint of a thermal transition temperature (melting temperature), Tm, was obtained from the DFS profiles. Protein folding was analyzed by examining circular dichroism (CD) spectra of protein samples. The spectra were scanned from 190‒200 nm with a CD spectrophotometer (J1700; JASCO, Tokyo, Japan). The obtained data were processed by using Origin 7 software. All the above tests were repeated three times.

## Results and Discussion

### The complex structure of EstL5-PMSF

**Figure 1:**
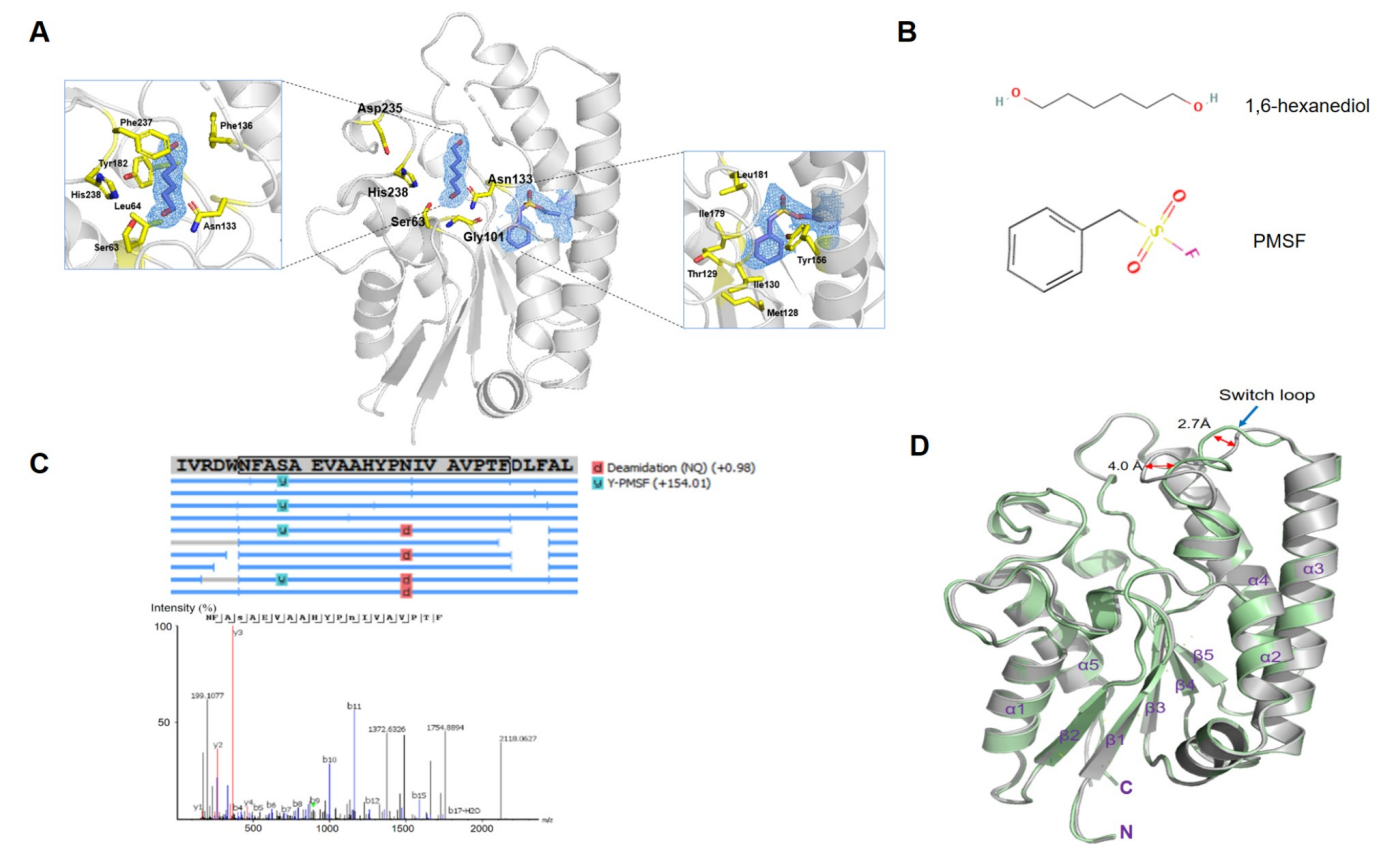
Figure 1 The overall structure of GDSL esterase EstL5 (A) The complex structure of Est5L with ligands. The 2Fo-Fc map is represented by a blue mesh contoured at 1.0σ, with the model including the ligands. Left panel: active site architecture of EstL5 with trapped 1,6-hexanediol, and right panel: surface cavity accommodated with covalently bound PMSF. Interacting residues are shown as stick representations. (B) Chemical structures of 1,6-hexanediol and PMSF. (C) Identification of the PMSF modification site on EstL5 by mass spectrometry. (D) Structural alignment between apo-EstL5 and EstL5-PMSF. The distances are given in angstroms. The r.m.s. deviation between them is 0.282 Å for main-chain atoms. Gray and green cartoons represent apo-EstL5 and EstL5-PMSF structure models, respectively.

Typically, the GDSL family esterases, as serine hydrolases, can be irreversibly inhibited by the fluorosulfate compound PMSF [14]. To analyze the catalysis of EstL5, we cocrystallized EstL5 esterase with PMSF. Through optimization experiments, the structure of esterase EstL5 in complex with PMSF was solved to 2.6 Å resolution. In the structure of EstL5-PMSL, two identical molecules were found in one asymmetric unit. The 2Fσ- Fc electron density contoured at the 1.0 σ level was well defined and continuous for the main chain and most of the side-chain residues in the model. After multicycle refinement, the Rfree and Rwork values decreased to 0.242 and 0.205, respectively. Interestingly, we observed an unexplained electron density in the vicinity of the active site of EstL5. By refining the model, the size and shape of the electron density indeed correspond to the solvent molecule 1,6-hexanediol ( Figure 1A, left panel). Furthermore, analysis of difference electron density maps also clearly revealed an additional electron density next to the hydroxyl oxygen of the noncatalytic serine residue Ser205. Unexpectedly, the PMSF molecule could be fitted nicely into the unmodelled electron density ( Figure 1A, right panel). The chemical structures of 1,6-hexanediol and PMSF are shown in Figure 1B. The distance to the density from the side chain of Ser205 indicates that this residue undergoes covalent modification by the PMSF moiety. To further confirm the bound PMSF groups, we employed nanoliquid chromatography coupled mass spectrometry to examine the obtained protein crystals. The PMSF-modified peptides were identified at the Ser205 residue of EstL5. Therefore, the PMSF group was verified to covalently bind to EstL5 at this noncatalytic site.

Similar to other GDSL esterases, the overall structure of EstE5-PMSF shows a typical SGNH hydrolase fold, consisting of five central parallel β-strands surrounded by five α-helices. The catalytic triad Ser63/Asp235/His328 and oxyanion hole Ser63/Gly101/Asn133, deduced by sequence homology, are situated on the concave surface of the enzyme. The structures of EstE5-PMSF and apo EstL5 are almost the same, as shown in the superimposition ( Figure 1B), with a low r.m.s. deviation value of 0.282 Å for main-chain atoms. The major conformation difference between the complex and apo EstL5 occurs at the switch loop (Gln137-Val146), which connects the β3 and α3 helices and contains Asn133 to stabilize the oxyanion intermediate during catalysis. Compared with the active site structure in the apo EstL5, the switch loop is shifted 2.6–4.0 Å down from an opening state towards a covering state above the active site ( Figure 1B). In fact, the presence of the bound 1,6-hexanediol in the active site of esterase EstL5 mimics the bound ester chain, which allows for further definition of its substrate pocket. We identified the residues Val168, Glu181, Tyr421, Ser420, Arg413, and Thr182, which are directly involved in the formation of the putative lipid binding pocket ( Figure 1A, left panel). This hydrophobic active site pocket seems to be large enough to accommodate medium-chain fatty acids but too small for long-chain fatty acids.

### PMSF binding cavity

**Figure 2:**
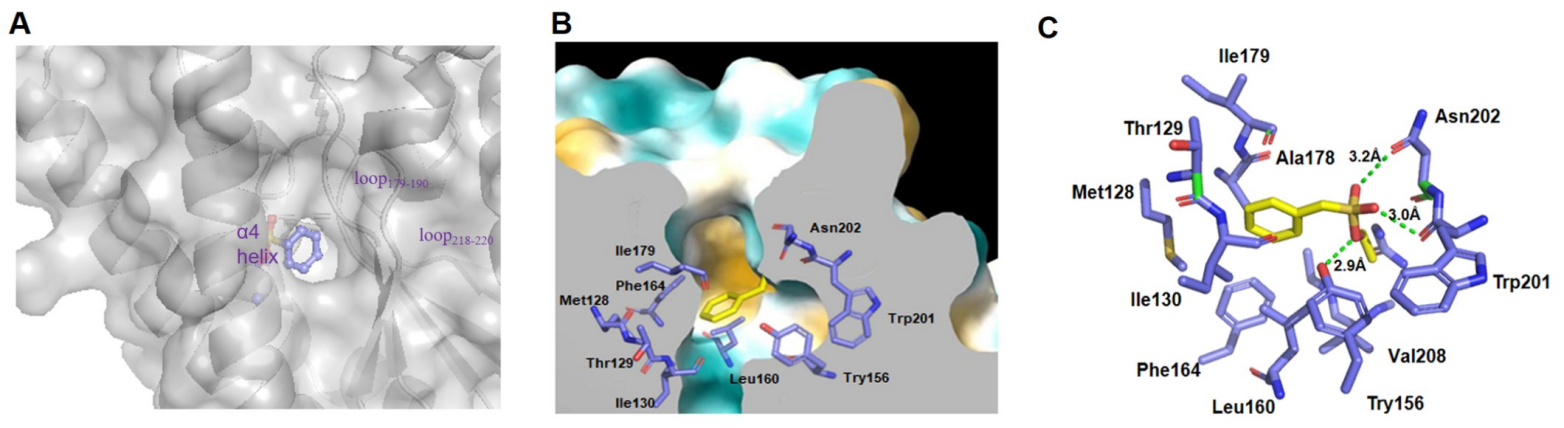
Figure 2 The surface cavity for accommodation of the PMSF moiety (A). The protein is shown as a gray surface, and the entrance of the PMSF accommodation cavity is located between helices α4, loop 218‒220 and loop 179‒190. The bound PMSF molecule is shown as colored spheres. (B). A surface representation of the cavity interior for PMSF accommodation, colored by the extent of hydrophobicity (blue, minimum; white, 0; orange, maximum). (C) Analysis of molecular interactions between EstL5 and PMSF. The PMSF moiety and possible interacting residues are shown as yellow sticks and purple sticks, respectively.

Because the enzyme’s active site offers hyperactivity of the catalytic serine residue, PMSF can specifically bind to the active site serine residue, but it seldom binds to other serine residues of proteins [8]. In the complex structure of EstL5-PMSF, we noted that PMSF is accommodated in a surface cavity, which has an entrance located between helices α4, loop _218-220_ and loop _179-190_ ( Figure 2A). Structurally, PMSF binds to Ser205, which is situated at the sidewall of the surface cavity, surrounded by relatively hydrophobic residues ( Figure 2B). The fact that the hydroxyl group of Ser205 is sulfonylated by PMSF suggested that this site Ser is a highly reactive residue. Indeed, the properties of residues in cavity usually differ from those on protein surface because surface cavities generally create specific microenvironments distinctive from bulk aqueous solutions [16]. In the EstL5 structure, neighboring residues, Tyr156 and Asn202, likely reduce the hydroxyl group pKa, contributing to the reactive activity of Ser205. A close-up view of PMSF with surrounding amino acid residues showed that Ser205 covalently binds to the sulfate center of PMSF, forming an o-benzylsulfonyl-serine ( Figure 2C). Furthermore, oxygen atoms of the sulfonyl group in PMSF interacts with the side chain of Tyr156 and the main chain of Trp201 and Asn202 at distances of approximately 2.9 and 3.0, 3.2 Å, respectively. The benzyl group of PMSF points toward the interior of the channel and is stabilized by a hydrophobic residue cluster, including Met128, Thr129, Ile130, Leu160, Phe164, Ala178 and Ile179 ( Figure 2C).

After comparing the residues surrounding PMSF with those in the apo EstL5 structure, we observed that the entry of PMSF does not lead to significant conformational changes in the surrounding amino acids. In the presence of PMSF, Tyr156, Leu181, and Asn202 display a slight shift, probably due to making room for PMSF binding. For EstL5, the presence of highly reactive Ser205 in the surface cavity also offers an ideal scaffold for engineering a new enzyme with multiple active sites [16]. Additionally, this specific serine from the protein surface cavity targeted by the fluorosulfate moiety suggested that it could be a suitable strategy for the development of covalent chemical modifications to noncatalytic serines of proteins.

### Identification of a unique channel leading to the active site pocket

**Figure 3:**
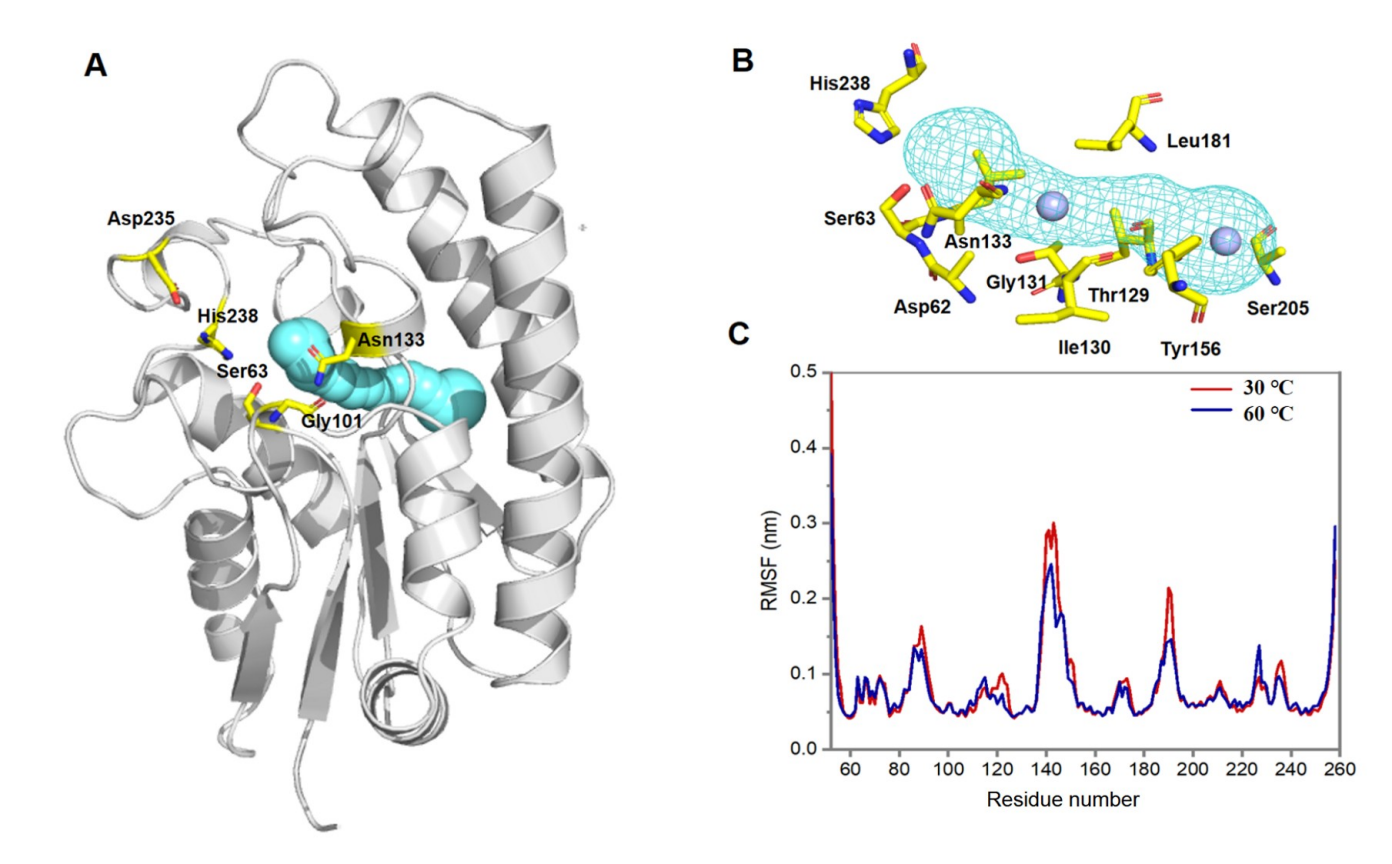
Figure 3 The unique channel identified in the EstL5 structure (A) A long channel was identified to lead through the matrix of the EstL5 enzyme, computed with CAVER 3.0. The channel is shown with a surface in cyan color, connecting the active site to a distal surface cavity. The protein is shown as a gray cartoon representation, and active site residues are shown as yellow sticks. (B) The channel in EstL5 is shown as a cyan mesh representation. Two water molecules at the channel interior are shown as spheres: one is situated at the surface entrance, and the other is located at the bottleneck of the channel. (C) Root mean square fluctuation (RMSF) of EstL5 enzyme backbones in 200 ns constrained MD simulation under different temperatures.

Notably, it was observed that the PMSF binding cavity defines a unique channel in EstL5 that clearly forms a connecting pathway between the active site and the distal surface cavity of the enzyme ( Figure 3A). Different from the tunnels, the channels of proteins construct void pathways, leading through the matrix of proteins, and both of their endings are opened to the surrounding solvent [17]. Recent studies have revealed that protein channels and tunnels have a significant impact on the activity, selectivity or stability of enzymes [ 18‒ 20] . To detect the topology of the channel, we used the CAVER tool [12] to calculate the access interior space in EstL5. The analysis results indicated that this unique channel in EstL5 extends approximately 18.9 Å, with a bottleneck radius of 6.8 Å, suggesting that it at least serves as an active site pocket branch and allows the surrounding solvents or small molecules to access the active site of EstL5. Indeed, we noted that two water molecules distributed at the beginning and middle of the channel. Those residues lining the channel were further identified, including Ser205, Tyr156, Thr129, Ile130, Gly131, Leu181, Asn133, Asp62, Ser63, and His238 ( Figure 3B), and this internal channel in EstL5 is mainly formed by aliphatic residues, which confer a relatively hydrophobic environment all along it.

Protein dynamics acts on various aspects of enzyme function [21]. For instance, side chain flipping or conformational alteration can reshape the internal space of proteins, which could obstruct or free the channels and tunnels that allow chemical molecules or solvents to migrate during enzyme catalysis. As reported earlier, EstL5 is a moderately thermostable GDSL family esterase, displaying catalytic activities within a relatively wide temperature range [7]. We further used molecular dynamic simulations to infer the detailed conformation motion features of EstL5 at different temperatures. At both 30°C and 60°C, the molecular dynamic simulations for 200 ns revealed significant fluctuations at residues 133 to 154 and 177 to 195 ( Figure 3D), which correspond to two highly flexible loop regions near the sidewalls of the access channel. The observed structural dynamics suggested that the local structural variability of the protein could affect the channel geometry in EstL5, which could facilitate molecule diffusion to and from the catalytic center.

### Structure-guided site-directed mutagenesis

Many protein channels or tunnels have been shown to play important roles in their function. Engineering of channel or tunnel internal residues offers higher chances of obtaining functional variants [17]. To explore the function of this channel in EstL5, we designed the replacement of the Ser205 residue by site-directed mutagenesis. By introducing the bulk aromatic sidechain residues Phe or Trp, the channel would be partially blocked at one end.

**Figure 4:**
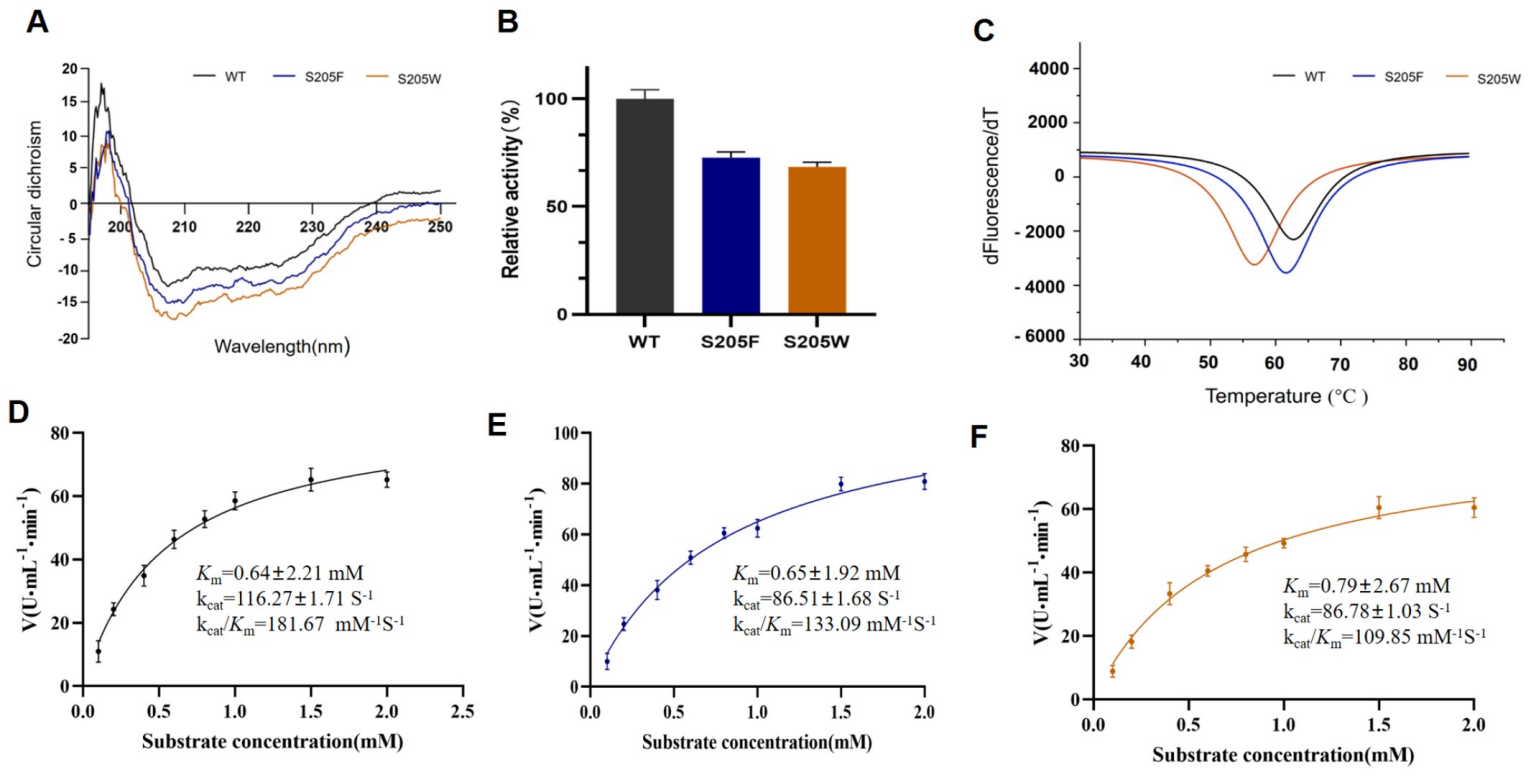
Figure 4 Biochemical analysis of Est5L mutants (A) CD spectra of wild-type EstL5 (WT) and mutants EstL5 S205F and EstL5 S205W recorded as protein folding analysis. CD data were obtained at a protein concentration of 0.1 mg/mL in 10 mM Tris-HCl buffer (pH 8.0), containing 0.1 M NaCl. (B) Enzyme activity examination of WT EstL5, EstL5 S205F and EstL5 S205W. (C) Analysis of enzyme thermodynamic stability. DSF was used to measure WT EstL5, mutant EstL5 S205F and EstL5 S205W proteins unfolding by monitoring changes in fluorescence as a function of temperature, and Tm values were calculated. (D‒F) Enzyme kinetic analysis of WT EstL5 and the EstL5 S205F and EstL5 S205W mutants using pNP-butyle as the substrate. The curve lines represent the data fitted to the Michaelis-Menten equation.

We compared the enzymatic activity of mutants *EstL5 S205F* and *EstL5 S205W* with that of WT at 30°C to assess mutation effects during catalysis. Circular dichroism spectra analysis showed similar peaks between the mutants and WT ( Figure 4A), indicating that the substitution of Ser with bulk aromatic residues in the channel did not affect protein folding. The resultant mutants, both *EstL5 S205F* and *EstL5 S205W*, caused a reduction in the catalytic activity of EstL5 ( Figure 4B). Furthermore, we performed steady-state kinetics analysis, and the Michaelis-Menten plots are presented in Figure 4D‒F. Although these two mutants displayed *K*_m_ values similar to that of WT, their turnover number ( *k*_cat_) value was 86.51±1.68 S ^‒1^ and 86.78±1.03 S ^‒1^, respectively, which is lower than the kcat of 116.27±1.71 S ^‒1^ for WT EstL5. Compared to wild-type enzymes, the *k*_cat_/ *K*_m_ of mutant *EstL5 S205F* and *EstL5 S205W* was obviously reduced. The decreased enzyme activity could be derived from protein internal pathway blocking, which would result in a limited transfer or diffusion of substrates, products and solvation along the channel during catalysis.

We also characterized the protein melting temperatures (Tm) of mutants and compared them with that of WT by thermofluor assays [12]. Logically, filling these protein interiors usually improves the hydrophobic packing of protein folds, therefore favoring a more stable conformation of enzymes. However, the results showed that the variant exhibited a Tm of 60°C, which is close to that of WT ( Figure 4C), indicating that these mutants contribute less to the thermodynamic stability of the enzyme. It is likely that the different profiles that have been observed highlight the difference in the filing capacity for each of the catalytic environments.

## Conclusion

In this study, we successfully solved the high-resolution X-ray crystal structure of the GDSL family member EstL5 complexed with PMSF. As a potent inhibitor of serine hydrolases, PMSF does not bind to the catalytic Ser63 residue in the complex structure. Unexpectedly, it was found that PMSF covalently binds to the noncatalytic Ser205 in a surface cavity. Structurally, a trapped crystallization solvent, 1,6-hexanediol, at the active site mimics the bound ester chain, which allows for further definition of the active site pocket of EstL5. Intriguingly, the presence of a unique long channel connecting the active-site pocket and the surface cavity mediates the transfer of substrates and products to and from the active site of the enzymes. By replacing Ser205 by a bulk aromatic residue Trp or Phe, the channel could be partially blocked at one end to perturb its access. Activity assays of the resultant mutants suggest the functional relevance of the channel to enzyme catalysis. This study further enhances our understanding of the catalytic properties of GDSL family enzymes and would help in the design of lipolytic biocatalysts for applications.
